# Fine-Scale Inference of Ancestry Segments Without Prior Knowledge of Admixing Groups

**DOI:** 10.1534/genetics.119.302139

**Published:** 2019-05-23

**Authors:** Michael Salter-Townshend, Simon Myers

**Affiliations:** * School of Mathematics and Statistics, University College Dublin, Ireland; † Dept. of Statistics, University of Oxford and Wellcome Trust Centre for Human Genetics, Oxford, UK

**Keywords:** population genetics, admixture, drift, selection, demography

## Abstract

We present an algorithm for inferring ancestry segments and characterizing admixture events, which involve an arbitrary number of genetically differentiated groups coming together. This allows inference of the demographic history of the species, properties of admixing groups, identification of signatures of natural selection, and may aid disease gene mapping. The algorithm employs nested hidden Markov models to obtain local ancestry estimation along the genome for each admixed individual. In a range of simulations, the accuracy of these estimates equals or exceeds leading existing methods. Moreover, and unlike these approaches, we do not require any prior knowledge of the relationship between subgroups of donor reference haplotypes and the unseen mixing ancestral populations. Our approach infers these in terms of conditional “copying probabilities.” In application to the Human Genome Diversity Project, we corroborate many previously inferred admixture events (*e.g.*, an ancient admixture event in the Kalash). We further identify novel events such as complex four-way admixture in San-Khomani individuals, and show that Eastern European populations possess 1−3% ancestry from a group resembling modern-day central Asians. We also identify evidence of recent natural selection favoring sub-Saharan ancestry at the human leukocyte antigen (HLA) region, across North African individuals. We make available an R and C++ software library, which we term MOSAIC (which stands for MOSAIC Organizes Segments of Ancestry In Chromosomes).

## Abstract (precis)

Salter-Townshend and Myers present an open source tool for modelling multi-way admixture events using dense haplotype data. Their Hidden Markov Model approach is scalable to thousands of samples and, unlike existing methods...

ADMIXTURE occurs when reproductive isolation between groups allows genetic divergence via genetic drift and random mutation, followed by mixing of the diverged groups to form new populations. Such genetic admixture is near ubiquitous in observed human populations (Patterson *et al.* 2012; Loh *et al.* 2013; Hellenthal *et al.* 2014) and indeed other species including cattle (Upadhyay *et al.* 2017), bison (Musani *et al.* 2006), and wolves (Pickrell and Pritchard 2012).

Genome-wide summaries can reveal not only complex relationships between modern populations but also details of their demographic histories (Pickrell and Pritchard 2012; Hellenthal *et al.* 2014; Peter 2016) while accurate inference of local ancestry can be used to correct for population structure in association testing (Diao and Chen 2012; Xu and Guan 2014), detect selection (Zhou *et al.* 2016), and can be used for mapping disease loci (Zhang and Stram 2014).

Due to the process of recombination, contiguous chunks of admixed individuals’ genomes are inherited intact from one mixing population or another. In the second generation following the initial admixture, chromosomes from distinct ancestral groups begin to recombine, and so the expected length of these chunks (in units of Morgans) will be 1 (by definition), and (neglecting crossover interference) chunk lengths can be modeled using an exponential distribution with rate parameter 1. In each subsequent generation, recombination further breaks down these chunks so that the chunk lengths (if they could be observed) are distributed according to an exponential distribution with rate parameter one less than the number of generations since admixture.

To fully characterize admixture for the above purposes, we need to infer: (1) Whether a group of individuals are admixed; (2) the component/mixing groups; (3) the timing of the admixture event(s); and (4) which segments of the admixed genome are inherited from each mixing group. Typically, we lack prior knowledge of each of these points and we do not have access to representative samples of the mixing groups, as these are often no longer present (without drift) in modern samples.

A wide variety of approaches to model admixture have been developed in recent years. STRUCTURE (Pritchard *et al.* 2000) clusters similar genomes together by fitting a mixture model using Gibbs sampling and STRUCTURE 2.0. Falush *et al.* (2003) extended this model to allow for admixed individuals using a Hidden Markov Model (HMM) that allowed for linkage along the genome. A drawback of these and similar approaches (*e.g.*, Sohn *et al.* 2012) is that they do not attempt to fully model linkage disequilibrium (LD), because SNPs within each source population are assumed to be independent, meaning they are not maximally powerful for inferring ancestry segments, particularly for subtle admixture events.

Other approaches focus on dating/characterizing admixture events, without performing local ancestry estimation. In the ALDER model (Loh *et al.* 2013) the exponential decay of ancestry segments is estimated as a function of genetic distance, allowing dating of admixture events. GLOBETROTTER (Hellenthal *et al.* 2014) uses a related approach for dating events by leveraging haplotype data, accounting for LD, but also infers admixture proportions and properties of the ancestral mixing groups, by quantifying their relationships with modern observed populations, and can handle multi-way admixture. In common with other approaches (some discussed below), GLOBETROTTER incorporates LD between nearby SNPs by fitting a haplotype copying model (Lawson *et al.* 2012) closely related to the Hidden Markov model introduced by Li and Stephens (2003). Here, “target” chromosomes of interest are formed as a mosaic whereby they imperfectly “copy” segments of DNA from donor haplotypes, according to a HMM. See Gravel (2012) for a review of this and other local ancestry models. The subsequent copying profiles (both global and locally along the genome) are analyzed and decomposed. Admixture times are then inferred by fitting curves measuring the correlation in copying along the genome: the relative probability of copying from pairs of donor populations is estimated at increasing genetic distances.

Several statistical algorithms [*e.g.*, MultiMix (Churchhouse and Marchini 2013), LAMP-LD (Baran *et al.* 2012), ELAI (Guan 2014), and HapMix (Price *et al.* 2009)], have been developed to identify local ancestry segments while accounting for LD at both the admixture and background scales. The first two rely on “windowing” the genome into nonoverlapping segments of equal size, and ancestry switches are assumed to occur only at window boundaries, whereas the second two build two-layer HMMs that allow ancestry switching anywhere along the genome. However, all of these rely on prespecification of the number of mixing groups, and the inclusion of groups of donor samples identical to, or at least closely related, to these mixing groups. HapMix models both pre and post-admixture recombination via a two-layer HMM, using an algorithm based on an extension to Li and Stephens (2003) and related to that developed here. However, HAPMIX allows for only two admixing groups and the user must supply known surrogates for both ancestries (although a low mis-copying rate is allowed for in the model to facilitate copying from the surrogates not associated with the current local ancestry).

A related, but different, approach is taken under the Conditional Sampling Distribution framework of Steinrücken *et al.* (2013). This approach considers a particular haplotype and tracks when, and with which other haplotype, it first coalesces in a HMM, *given* an underlying demographic history of the samples. Thus hypotheses of historic migration, etc., may be tested using the samples. This was further extended to model admixture in Steinrücken *et al.* (2018), with a specific application in detecting the introgression of Neanderthal tracts into non-African genomes. This model, DICAL-ADMIX, requires first fixing the demographic model [although demographic inference is demonstrated in the related model of Steinrücken *et al.* (2015)] and then estimating gene-flow rates, times, etc. Spence *et al.* (2018) discusses these models in the context of extensions to Li and Stephens (2003), and notes that the “method can scale to tens of haplotypes and has been used on models with three populations, but can handle arbitrarily many populations at increased computational cost.”

LAMP-LD (Baran *et al.* 2012), RFMix (Maples *et al.* 2013), and ELAI (Guan 2014) are among the most widely known local ancestry methods. The LAMP-LD model was created to model local ancestry in Latino populations, comprising a HMM copying model that is optimized for recent three-way admixture for which good surrogate donor reference panels are available. It can outperform other approaches even for two-way local ancestry inference in certain settings (*e.g.*, African-American simulated admixture six generations ago). This is achieved by building a two-layer HMM with the first layer defined on nonoverlapping, evenly sized, windows. Ancestry switches within windows do not occur, but ancestry switches are allowed at the window boundaries. Conditional on hidden ancestry states on the ends of a window, the genotypes are emitted by pairs of the second layer HMMs. The most likely pair of local ancestries across the windows is inferred before a postprocessing lifting on the restriction of ancestry switches at boundaries. This equates to a reduced state-space in the first instance, followed by a pass with a full set of states. As noted in Guan (2014), the largest concern is the overconfidence of the method, manifesting as very certain local ancestry assignment almost everywhere. Crucially, as per the other methods listed here, a close correspondence between ancestral mixing groups and donor reference panels is both required and assumed known—an assumption we do not make. The method does not estimate parameters such as generations since admixture, and assumes that all individuals have experienced the same admixture event in terms of proportion and date.

RFMix differs from the other methods listed here in that a discriminative, rather than generative, approach is used to infer local ancestry tracts. Again, a known close mapping between latent ancestral and observed surrogate (reference) donor sets is required. Equally sized windows along chromosomes are then used, and, within each window, a random forest is trained to infer ancestry using the reference panels. A HMM is then trained to output the smoothed local ancestry estimates along the entire chromosome. The method scales well, and can handle multiway admixture scenarios. Similarly to our approach, phasing errors within the admixed samples can be corrected; however, RFMix assumes that there is, at most, one such error per ancestry window to correct, whereas we do not assume an upper limit on the number of these errors. Demographic parameters such as generations since admixture are not inferred by the method but related settings (window size in centi-Morgans) must be provided.

ELAI works with diploid admixed samples (reference panels should be phased), but can make use of phased haplotypes if available. No recombination map is required as local recombination rates are implicitly estimated. However, this can lead to reduced accuracy in settings where the reference panels are small, or it is otherwise difficult to estimate the recombination rates this way. Like HapMix, ELAI can detect short local ancestry tracts (a few tenths of a centi-Morgan). By assigning weights to cohort samples it can be applied to large samples and weights are taken such that the effective sample size for each ancestry is twice the target sample size. One advantage of ELAI is that it does not require *ad hoc* division of the chromosomes into equally sized windows of single ancestry. As per all other existing local ancestry methods that we are aware of, ELAI requires training panels that represent good direct surrogates for the mixing populations, and, thus, does not infer the panel-ancestry relationship. It can be run without surrogates for one of the mixing groups (as noted in Zhou *et al.* 2016) and requires a single admixture date as input.

We compare results on simulated three-way admixture using LAMP-LD, ELAI, RFMix, and MOSAIC in the Supplemental Material, Section S5, noting that MOSAIC has uniformly superior accuracy to the other approaches for our test cases. For the simulations, we admix real chromosomes from Europe, Asia, and Africa based on varying generations since mixing. We then infer local ancestry using each method and reference haplotypes from the three continents that do not include individuals from the populations used to create the admixed samples. The unique contribution of MOSAIC is that specification of the potentially complex relationships between reference haplotypes and ancestral mixing groups is not required, unlike all other methods. Even when this knowledge is provided to the other methods, MOSAIC is able to achieve the highest correlation with true local ancestry (see Table S4). As Wangkumhang and Hellenthal (2018) note, this is mainly due to the fact that “one notable limitation is that most approaches rely on using surrogates for the original (unknown) admixing sources, and it is unclear how accurate these surrogates may be. For example, often modern-day samples are used as surrogates despite themselves having recent admixture from other sources.” MOSAIC does not rely on such direct surrogates, but learns the indirect relationships between the latent ancestral groups and the panels of donor haplotypes from the data.

## MOSAIC Overview

In our approach, the unseen mixing source populations are decoupled from the observed reference populations (of which there may be many), and the details of relationships between the two are inferred as part of the algorithm. Our approach takes one or more perhaps admixed genomes, compares to previously labeled (*e.g.*, by region of origin, or genetic clustering) groups/reference panels of additional individuals, and identifies and characterizes segments of local ancestry for admixture of arbitrary numbers of populations. Note that we do not require that the unobserved admixing ancestral groups to be a close match to the observed labeled groups, but, rather, we learn the genetic relationships between them. We exploit LD information to decompose the genome into segments, and use an HMM algorithm, similar in spirit to that of HapMix, which forms a special case. Each admixing population copies from each panel according to a set of weight parameters inferred by the method. For example, in Hazara two-way admixture, we find that individuals possess admixture segments from two groups, one preferentially copying from donors that are North East Asian, and one from Central/South Asian donors, matching previous findings (*e.g.*, Hellenthal *et al.* 2014).

To avoid phasing errors scrambling ancestry switch signals within the inferential algorithm, we iteratively update haplotypic phase [a similar approach, although with a different underlying algorithm, to that used in *e.g.*, RFMix (Maples *et al.* 2013)], and infer time since admixture via the fitting of exponential decay coancestry curves. We estimate the drift between the unobserved ancestral groups and other groups by constructing partial genomes from the admixed individuals themselves, representative of the original nonadmixed ancestral individuals. These can then be compared to other populations, allowing estimation of divergence as ${\text{F}}_{\text{st}}$.

Our software—MOSAIC—returns parameter estimates, local ancestry estimates along the genome, coancestry curves (including the best fit exponential curve corresponding to estimated pairwise ages of admixture), ancestry informed phase of the target haplotypes, and ${\text{F}}_{\text{st}}$ estimates between the ancestral mixing groups and between the mixing groups and each panel.

## Materials and Methods

The inputs are haplotypes from labeled subpopulations (panels), target admixed haplotypes, and recombination rate maps. Typically, the phasing of the haplotypes is accomplished algorithmically [here, we have used SHAPEIT2 (Delaneau *et al.* 2013)]. Within our inferential algorithm, we allow for, and attempt to correct, phasing errors in the admixed targets.

### Overview of the model

**Figure 1:**
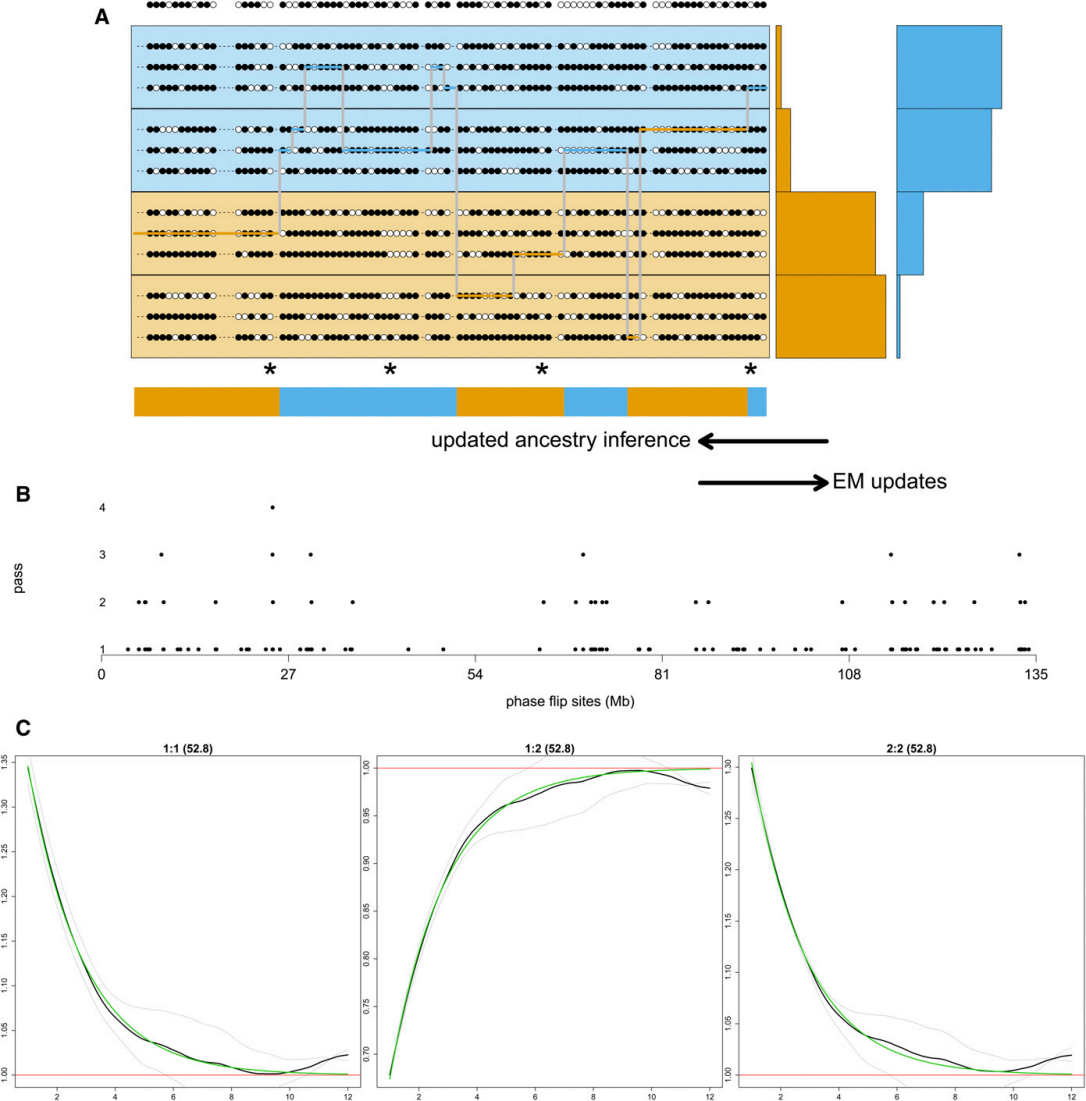
MOSAIC proceeds by rounds of thin (see *Thinning*), EM (see *EM Updates*), phasing (see *Rephasing*). (a) is a cartoon version and (b and c) depict the simulations used to test the approach in Simulation Studies. (a) The top row is a single observed admixed haplotype. The four panels beneath it each have three reference haplotypes, in this case separable into two diverged groups (orange and blue). Local ancestry estimates (colors along the bottom) are estimated, conditional on parameter estimates including the conditional probability of selecting a panel given the local ancestry (right hand side). Estimated local ancestry is then used to update parameter estimates in an EM algorithm. A key innovation here is demonstrated by the segment second from the right, wherein a putatively blue haplotype is copied under an orange ancestry. Filled and open circles denote reference and alternative allelic types, and the asterisks denote miscopied alleles. (b) The phase-hunter method applied to a simulated admixed chromosome 10. The dots show the locations along a chromosome (*x*-axis) that are flipped for phase by the algorithm at successive rounds of the phase-hunter (*y*-axis). Fewer sites are candidates (increased log-likelihood if flipped) in each round. Just four forward-backward algorithm passes are required to find all single phase flips that increase the log-likelihood in this example. (c) Dating is estimated using the coancestry curve fitting in *Dating Admixture Events Using Coancestry Curves* using the exponential decay of the ratio of probabilities of pairs of local ancestries (*y*-axis) as a function of genetic distance (in centi-Morgans, *x*-axis). The green line depicts the fitted curve, the black line the across targets observed ratios, and the grey lines the per target ratio. Along the top of each panel is the index of the pair of ancestries being examined as a:b followed by the estimated decay parameter in brackets corresponding to the number of generations since admixture. In this case, 50 generations since admixture has been simulated and we demonstrate in Section S2.1 of the Supplement that bootstrapped samples (see *Simple two-way admixture analysis*) of the inferred date are centered around this value.

Figure 1 outlines our model in graphical form, with further details below. Figure 1a depicts the phased part of our approach, while we describe phasing steps in *Rephasing* and Figure 1b. Figure 1a shows 12 phased haplotypes sampled from four labeled populations (panels). The target haplotype is the result of two-way admixture from unknown ancestral *source* groups. The labeled populations act as *surrogates* for these sources. However their relationships with the source groups is not known in advance, but learned from the data. We model the target haplotype as a mosaic, copying segments from members of the labeled populations, via a HMM related to that of Li and Stephens (2003). A matrix of *copying probabilities* (right side of Figure 1a parameterises how the surrogates relate to the underlying ancestry, and this matrix is learned from the data to characterize the admixing source groups. If a panel does not contain useful surrogate haplotypes for the mixing groups, then the corresponding row of the matrix will be near zero. Conversely, if several panels contain good surrogates for an ancestry, then they will share the conditional probability mass with obvious postfit interpretation. Finally, if there is a panel that is similarly admixed to the target individuals, this will be reflected as a row in the copying matrix with multiple nonzero entries. The hidden states in our HMM consist of two layers: each site (or gridpoint, see *Gridding on genetic distance*) has both a hidden local ancestry (blue or orange in the Figure) and another hidden state specifying which donor haplotype is being copied from.

### Two-layer HMM

Our approach may be viewed as a combination of HapMix (Price *et al.* 2009) and GLOBETROTTER (Hellenthal *et al.* 2014). As per HapMix, admixture is directly incorporated into the HMM. Unlike HapMix, our model works with any multiple of ancestry sources^1^ and is more flexible; not only do we allow for a rich variety of dependency between latent ancestral sources and labeled modern populations, but we also do not require prespecification of these dependencies (see *Transitions* for how we parameterize these relationships). As in GLOBETROTTER, MOSAIC infers the relationship between modern populations and ancient unseen mixing populations from the data. The key difference is that our method builds these relationships directly into the HMM, which uncovers accurate local ancestry estimates along the genome, whereas GLOBETROTTER fits a mixture model to the output of an ancestry unaware HMM.

### Gridding on genetic distance

We impose an even grid on recombination distance along each chromosome, to speed up the algorithm (we use fewer gridpoints than SNPs), and simplify HMM calculations (recombination distances are constant on the grid). SNPs are mapped to their nearest gridpoint, according to genetic distance. This is extremely fine (60 gridpoints per centimorgan) in contrast with the single-ancestry windows imposed by existing admixture models such as MultiMix (Churchhouse and Marchini 2013) and LAMP-LD (Baran *et al.* 2012). We make the simplifying approximation that recombination only happens between successive gridpoints, which will be accurate for the time frame on which we focus and greatly simplifies the mathematics of our inference. There can now be 0, 1, or multiple SNPs at any given gridpoint, and the HMM is defined at all gridpoints. The emission probabilities (see *Emissions*) for a gridpoint and potential donor haplotype depend only on the number of matching and nonmatching SNPs for that gridpoint and donor, which are calculated once and stored. Phasing is also calculated only at gridpoints. The density of markers does not impact the speed of our inference, aside from the initial additional overhead in reducing the data to the grid, meaning that MOSAIC scales sublinearly with the number of sites.

### Transitions

We jointly model ancestry and haplotype, copying chunks along the genome using a two-layer HMM. The first layer involves ancestry switches along the genome, and the second layer switches between copied haplotypes along the genome. Ancestry switches occur at a slower rate than the haplotype switches as they only occur postadmixture and each ancestry switch enforces a haplotype switch in the model. The probability of making a switch from ancestry *b* to ancestry *a* between successive gridpoints is parameterised in our model as $\Pi_{ba}^{\left(n\right)}$ in target individual *n*. Note that this individual specific A×A matrix (where *A* is the number of latent ancestries) of switch rates encompasses all that the model knows about the admixture event, *i.e.*, the time of the event and the mixing proportions, and is not constrained to be symmetric.

The probability of switching to any donor haplotype depends on the size of the panel, and the underlying local ancestry at the gridpoint. We parameterise by $\mu_{pa},$ the probability of selecting from panel *p* when the local ancestry is *a* (*i.e.*, the *copying probabilities* on the right side of Figure 1a). The columns of μ are constrained to sum to unity, and we scale these probabilities when used in our HMM by $N_{p}$, the number of donor haplotypes in panel *p*. Finally, we denote the recombination within ancestry probability with *ρ*, *i.e.*, this is the recombination rate conditional on no ancestral switch.

The transition probability of making a switch from (ancestry, haplotype) pair $\left(b,h_{q}\right)$ to $\left(a,h_{p}\right)$ where $h_{p}$ is a donor haplotype *h* in panel *p* for target individual *n*, is given by:

$$\begin{array}{cc} \Pi_{ba}^{\left(n\right)}\frac{\mu_{pa}}{N_{p}} & a\neq b\\ \left(\left(1-\Pi_{a\cdot }^{\left(n\right)}\right)\rho +\Pi_{aa}^{\left(n\right)}\right)\frac{\mu_{pa}}{N_{p}} & a=b,h_{p}\neq h_{q}\\ \left(\left(1-\Pi_{a\cdot }^{\left(n\right)}\right)\rho +\Pi_{aa}^{\left(n\right)}\right)\frac{\mu_{pa}}{N_{p}}+\left(1-\Pi_{a\cdot }^{\left(n\right)}\right)\left(1-\rho \right) & a=b,h_{p}=h_{q}, \end{array},$$

where $\Pi_{a\cdot }^{\left(n\right)}=\sum_{b}\Pi_{ab}$.

Note that the $\Pi^{\left(n\right)}$ terms are unconditional probabilities (thus we do not multiply by *ρ*) and parameterise ancestry switches, but not nonswitches, so the sum of the rows is not constrained to be 1. The $\Pi^{\left(n\right)}$ matrices are specific to the admixed target individuals whereas μ and *ρ* are assumed to be common to the entire admixed population. Other choices are available to our model (and code) and can prove useful; for example, when a subset of admixed targets have undergone a markedly different admixture event to the rest, they should be modeled separately, resulting in two sets of parameters. The interpretation of the above choice is that the admixture is well characterized as being the mixing of a single set of well-defined ancestral populations, but each target individual may have experienced the mixing at a different point in their history (and in a different ratio). The default individual-specific $\Pi^{\left(n\right)}$ can be changed to a single joint Π, which equates to an assumption that each admixed genome has a shared admixture profile. This choice is suitable in the presence of panmixia postadmixture and an old admixture event. Where this assumption holds, similar estimates for each $\Pi^{\left(n\right)}$ result. In this work, we assume a single scalar *ρ*; we have experimented with ancestry specific versions of this parameter, but the vector ρ is then confounded with $\Pi^{\left(n\right)}$.

### Emissions

We deal with biallelic SNP data (denoted with a Y), and we use *θ* to parameterize the emission probability of a 1 at locus *l* when copying donor haplotype *h* as

$$\theta \left(1-Y_{lh}\right)+\left(1-\theta \right)Y_{lh},$$

where $Y_{lh}=1$ if donor haplotype *h* has biallelic SNP 1 at locus *l*, else it is 0. Thus, *θ* is the probability of a pointwise discrepancy between the allele of the haplotype being locally copied and the allele of the copying haplotype, *i.e.*, the miscopying rate. Note that, for notational simplicity, we have suppressed here the index of the panel from which that haplotype comes. As panel being copied does not impact our calculation, we could allow this to account for genetic drift between the ancestral groups and the modern reference panels; however, we do not believe it would result in large improvements in inferences based on exploratory analyses.

As we have moved the observed markers to a grid, each gridpoint may have zero, one, or multiple emissions (observations). This is handled simply by assuming a product of emission probabilities for multiple observations (or a sum over the log-probabilities in practice). For gridpoints with no observations, the emission probability is simply 1. This has the additional benefit of allowing our model to handle missing data, as there is simply a lower count of observations at a gridpoint when SNPs are missing.

### Algorithm

Our inferential algorithm comprises initialization of all parameters (see Appendix Initialization), followed by a loop over successive rounds of thinning (see *Thinning*), rephasing (see *Rephasing*), and EM updates of the parameters (see *EM updates*). We find that a low number (five for results presented here) of rounds of these three parts results in convergence to a final phasing solution. Within each round, we perform 10 EM iterations, with an additional final EM algorithm run until convergence.

1. Initialization of all parameters: μ,ρ,θ, Π.
2. Repeat until phase convergence:
  - thin.
  - rephase.
  - 10 EM iterations.
3. Final EM until convergence.
4. Coancestry curve fitting to estimate dates.

### Thinning

Thinning refers to a local (gridpoint specific) reduction of the set of possible donors available to copy for each target individual, and is a computationally convenient approximation. We fit a single-layer ancestry unaware model [similar to chromopainter (Lawson *et al.* 2012)] to the full set of donors. For each target individual, we then rank the donors at each gridpoint, and pass only the top 100 to the ancestry-aware two-layer HMM part of our model. For large reference datasets, this greatly reduces the state-space of the model with a negligible reduction in accuracy, as, typically, only a handful of donor haplotypes are likely to be copied from. The reason we do this per individual rather than per target haplotype is to make the donors relevant to both haplotypes available for the rephasing part of the algorithm. See *Appendix app:thinning* for full details.

### Rephasing

Phasing errors can potentially mask real ancestry switches, or cause our model to infer ancestry switches where there are none. HapMix solves this issue by integrating over all possible phasing at each locus (diploid mode), but this is computationally intensive and HapMix cannot run this option in conjunction with EM inference of the model parameters. To consider all possible phasing is intractable as we would need to consider $2^{G}$ possible phasings per individual genome, where *G* is the number of gridpoints. Instead, we search for phase flips that would lead to an increase in the likelihood of the data under our model, and hill climb to a maximum likelihood solution (“phase-hunting”). Although our software can also sample via MCMC other phase solutions, we find that our hill climbing method is both fast and leads to a log-likelihood that is not well improved upon by a long run of the MCMC chain. We use a pass of our fast forward-backward algorithm to see the *marginal* change in log-likelihood under the model were we to flip each gridpoint independently and examination of these log-likelihood changes informs our phase-hunter as follows:

1. Identify intervals for which phase flips give positive values in this expected log-likelihood change.
2. Flip all of the highest nonoverlapping (farther than 0.1 cM apart) intervals and refit our HMM.
3. Repeat this process as long as the log-likelihood increases.

In practice, we find fewer attractive phase-flips in each successive pass; see Figure 1b. Figure S3a and Section S3 demonstrate the contribution of this rephasing step to the overall model fit in comparison with HapMix (which integrates over all possible phasings) in the context of a two-way admixture problem.

### EM updates

The EM algorithm (also referred to as Baum-Welch when used in conjunction with a HMM) performs estimation of the hidden states given a set of parameter estimates (E-step), and then performs maximum likelihood estimation for the model parameters given these estimates (M step). Iteration then proceeds over the E and M steps until convergence to a steady set of parameters and local ancestry estimates. At each step the log-likelihood for the model is guaranteed to increase (although convergence to the global maximum is not guaranteed).

### Expected coefficient of determination

For simulated data, we report a measure of the accuracy of MOSAIC’s local ancestry with the commonly used measure of the squared sample correlation $r^{2}$ between the estimated local ancestry X (where $X_{ag}$ is the probability that the haplotype belongs to ancestry *a* at location *g* on the genome) and the true local ancestry Z. This is known as the Coefficient of Determination. However, in the absence of such a ground truth Z for real data, we use an expectation of this coefficient, as per Price *et al.* (2009).

For a given ancestry, *a*, we compute the expected value of this based on the inferred local ancestry, $X_{a}$, without knowledge of the true local ancestry, $Z_{a}$, for each individual as follows:

$$\begin{array}{ll} E\left[r^{2}\left(X_{a},Z_{a}\right)\right]=E\left[\frac{\text{cov}{\left(X_{a},Z_{a}\right)}^{2}}{\text{var}\left(X_{a}\right)\text{var}\left(Z_{a}\right)}\right]\\ & ≃\frac{E{\left[\text{cov}\left(X_{a},Z_{a}\right)\right]}^{2}}{\text{var}\left(X_{a}\right)E\left[\text{var}\left(Z_{a}\right)\right]}, \end{array}$$ (1)

where the expectations are with respect to the random variables $Z_{a}$. Given inferred $X_{a}$, the $Z_{ag}$ are assumed Bernoulli distributed with $P\left(Z_{ag}=1\right)=X_{ag}$. The sample variances are covariance are computed across gridpoints on the whole genome g=1…G.

To estimate the terms in Equation 1, we could take independent samples of $Z_{ag}$ based on $X_{ag}$ and use the sample mean of Equation 1 evaluated on each sampled $Z_{a}$ as an estimator for the expectation; however, we can directly estimate what each term will be. The sample variance of $X_{a}$ over gridpoints is directly given by:

$$\text{var}\left(X_{a}\right)=\frac{\sum_{g}\left(X_{ag}^{2}\right)}{G}-{\left(\frac{\sum_{g}X_{ag}}{G}\right)}^{2}.$$

The expected covariance between $X_{a}$ and $Z_{a}$ is given by

$$\begin{array}{l} E\left[\text{cov}\left(X_{a},Z_{a}\right)\right]=E\left[\frac{\sum_{g}X_{ag}Z_{ag}}{G}-\frac{\sum_{g}X_{ag}}{G}\frac{\sum_{g}Z_{ag}}{G}\right]\\ =\frac{\sum_{g}X_{ag}E\left[Z_{ag}\right]}{G}-\frac{\sum_{g}X_{ag}\sum_{g}E\left[Z_{ag}\right]}{G^{2}}\\ =\frac{\sum_{g}\left(X_{ag}^{2}\right)}{G}-{\left(\frac{\sum_{g}X_{ag}}{G}\right)}^{2}. \end{array}$$

which we note is the same as $\text{var}\left(X_{a}\right)$. The expected value of the variance of $Z_{a}$ is:

$$\begin{array}{l} E\left[\text{var}\left(Z_{a}\right)\right]=E\left[\frac{\sum_{g}Z_{ag}^{2}}{G}-{\left(\frac{\sum_{g}Z_{ag}}{G}\right)}^{2}\right]\\ =\frac{\sum_{g}E{\left[Z_{ag}\right]}^{2}+\text{var}\left(Z_{ag}\right)}{G}-{\left(\frac{\sum_{g}E\left[Z_{ag}\right]}{G}\right)}^{2}\\ =\frac{\sum_{g}X_{ag}^{2}+X_{ag}\left(1-X_{ag}\right)}{G}-{\left(\frac{\sum_{g}X_{ag}}{G}\right)}^{2}\\ =\frac{\sum_{g}X_{ag}}{G}-{\left(\frac{\sum_{g}X_{ag}}{G}\right)}^{2} \end{array}$$

Finally, cancelling terms, we get

$$E\left[r^{2}\left(X_{a},Z_{a}\right)\right]≃\frac{\sum_{g}X_{ag}^{2}-{\left(\sum_{g}X_{ag}\right)}^{2}/G}{\sum_{g}X_{ag}-{\left(\sum_{g}X_{ag}\right)}^{2}/G}.$$

This is then averaged over all ancestries *a* to return the expected squared correlation between the inferred local ancestry and the *unobserved* true local ancestry.

For diploid local ancestry, the haploid $X_{ag}$ is replaced by the sum of these over the two chromosomes for an individual, say $X_{ag}=X_{ag}^{\left(1\right)}+X_{ag}^{\left(2\right)}$, where the superscript indexes the two chromosomes. Each $Z_{ag}$ is then assumed to be the sum of two independent Bernoulli random variables with parameters $X_{ag}^{\left(1\right)}$ and $X_{ag}^{\left(2\right)}$. The expected value and variance of such a random variable is given by $X_{ag}$ and $X_{ag}-X_{ag}^{2}+2X_{ag}^{\left(1\right)}X_{ag}^{\left(2\right)},$ respectively. The above calculations for the variance of $X_{a}$ (which takes values 0–2) and the expected covariance between this and the diploid true ancestry $Z_{a}$ are unchanged, but the expected variance of $Z_{a}$ is then given by

$$E\left[\text{var}\left(Z_{a}\right)\right]=\frac{X_{ag}+2X_{ag}^{\left(1\right)}X_{ag}^{\left(2\right)}}{G}-{\left(\frac{\sum_{g}X_{ag}}{G}\right)}^{2}.$$

For the diploid local ancestry, we therefore get

$$E[r^{2}\left(X_{a},Z_{a}\right)]≃\frac{\sum_{g}X_{ag}^{2}-{\left(\sum_{g}X_{ag}\right)}^{2}/G}{\sum_{g}\left(X_{ag}+2X_{ag}^{\left(1\right)}X_{ag}^{\left(2\right)}\right)-{\left(\sum_{g}X_{ag}\right)}^{2}/G}$$ (2)

### $F_{\mathrm{st}}$ summaries

We use ${\text{F}}_{\text{st}}$ to summarize the genetic divergence of the mixing groups and further characterize each admixture event. As samples of the mixing groups are not available, we first assign *locally* segments of the target chromosomes to the ancestry; they are maximally assigned *a posteriori* based on the HMM fit.^2^ These partial, haploid genomes may now be thought of as drifted versions of the ancestral groups that mixed to create the targets. We then calculate ${\text{F}}_{\text{st}}$ (see below) between these so created “unadmixed” genomes and each panel (donor population) used in the model fit. If each panel represents good surrogates for one (and only one) unmixed ancestral group, then the ${\text{F}}_{\text{st}}$ between these partial genomes and the donor panels will be strongly negatively correlated with the corresponding elements of μ. If there is a panel that is comprised of individuals from a similar population to the target admixed population, then it will have high ${\text{F}}_{\text{st}}$ to both ancestral groups (and thus to these partial genomes), assuming accurate local ancestry deconvolution.

### $F_{\mathrm{st}}$ calculations

Noting that naïve estimators of ${\text{F}}_{\text{st}}$ may be biased, and that there are many estimators in the literature, we refer to Bhatia *et al.* (2013) to inform our choice. We follow the recommendations of that paper and use a ratio of averages (rather than an average of ratios) as this is numerically stable. To aggregate across loci, we first define SNP *s* specific ${\text{F}}_{\text{st}}^{\left(s\right)}$ as the estimated variance in the frequency between populations (weighted by population size), divided by the estimated variance of frequency across populations. We can then calculate the genome wide ${\hat{F}}_{\text{st}}$ ratios by summing the numerator and denominator across the genome first and taking the ratio.

Note that we deviate from the recommended choice of estimator for the numerator and denominator in Bhatia *et al.* (2013). They recommend the Husdon estimator (Bhatia *et al.* 2013, Equation 5) as this provides unbiased estimates for both the numerator and denominator but assumes equal and large sample sizes. As our sample size for the ancestral groups (constructed as above using maximal assignment^3^ of local segments of admixed genomes) varies along the genome, we prefer an estimator that is robust to sample size variation and can aggregate site specific contributions to the numerator and denominator with varying sample sizes. We therefore use the Weir and Cockerham (1984) estimator in a form that uses a ratio of averages. Specifically we use a variation of equation 6 in Bhatia *et al.* (2013):

$${\hat{F}}_{\text{st}}=1-\frac{\sum_{s=1}^{S}2a_{s}b_{s}c_{s}}{\sum_{s=1}^{S}d_{s}+\left(2a_{s}-1\right)b_{s}c_{s}},$$ (3)

where *s* is the site index, and

$$\begin{array}{l} a_{s}=\frac{n_{1s}n_{2s}}{n_{1s}+n_{2s}},\\ b_{s}=\frac{1}{n_{1s}+n_{2s}-2},\\ c_{s}=n_{1s}\left[{\hat{p}}_{1s}\left(1-{\hat{p}}_{1s}\right)\right]+n_{2s}\left[{\hat{p}}_{2s}\left(1-{\hat{p}}_{2s}\right)\right],\\ d_{s}=a{\left({\hat{p}}_{1s}-{\hat{p}}_{2s}\right)}^{2}. \end{array}$$

Note that *a* and *b* are unrelated to the *a* and *b* we used to index ancestries; here, we are using the same notation as Bhatia *et al.* (2013) for clarity. ${\hat{p}}_{is}$ is the observed allele frequency at site *s* for population *i*. The sample size for population *i* is $n_{is},$ and is fixed for the donor panels but varies for the partial ancestral group genomes created from the admixed haplotypes.

We also calculate ${\text{R}}_{\text{st}}=\text{R}\left({\hat{F}}_{\text{st}}\right)$ as the estimated average-over-panels relative squared difference in ${\text{F}}_{\text{st}}$ between ancestral groups *a* and *b* to each panel as a measure of whether the panels are useful in differentiating the groups:

$${\text{R}}_{\text{st}}\left(a,b\right)=\frac{1}{P}\sum_{p=1}^{P}\frac{{\left({\hat{F}}_{\text{st}}\left(a,p\right)-{\hat{F}}_{\text{st}}\left(b,p\right)\right)}^{2}}{0.5\left({\hat{F}}_{\text{st}}\left(a,p\right)+{\hat{F}}_{\text{st}}\left(b,p\right)\right)}.$$ (4)

Thus, ${\text{R}}_{\text{st}}$ is the mean across panels measure of the ratio of the squared difference in genetic divergence between the panel and the ancestral groups to the sum of the genetic divergences between panel and ancestral groups. Only panels that are diverged by dissimilar degrees contribute, and ${\text{R}}_{\text{st}}$ varies between 0 and 2.

${\text{R}}_{\text{st}}$ is correlated with ${\hat{F}}_{\text{st}}$ between the latent ancestries (Pearson correlation of 0.23 with *P*-value of 0.00047 across all 95 real populations we analyzed); however, it also takes into account the distances to the donor groups. For example, the mixing groups could be far apart, as reported by ${\hat{F}}_{\text{st}};$ however, the panels may be poor surrogates due to drift since admixture. This causes each panel to have a similar ${\hat{F}}_{\text{st}}$ to both ancestral groups, and this will show in a low ${\text{R}}_{\text{st}}$ overall. The correlation between ${\text{R}}_{\text{st}}$ and $E[r^{2}]$ was 0.3 with a *P*-value of $3.2\times 10^{-6},$ whereas the correlation between ${\hat{F}}_{\text{st}}$ and $E\left[r^{2}\right]$ was 0.057 with a *P*-value of 0.4, showing that ${\text{R}}_{\text{st}}$ is the better indicator of accuracy of local ancestry.

**Figure 2:**
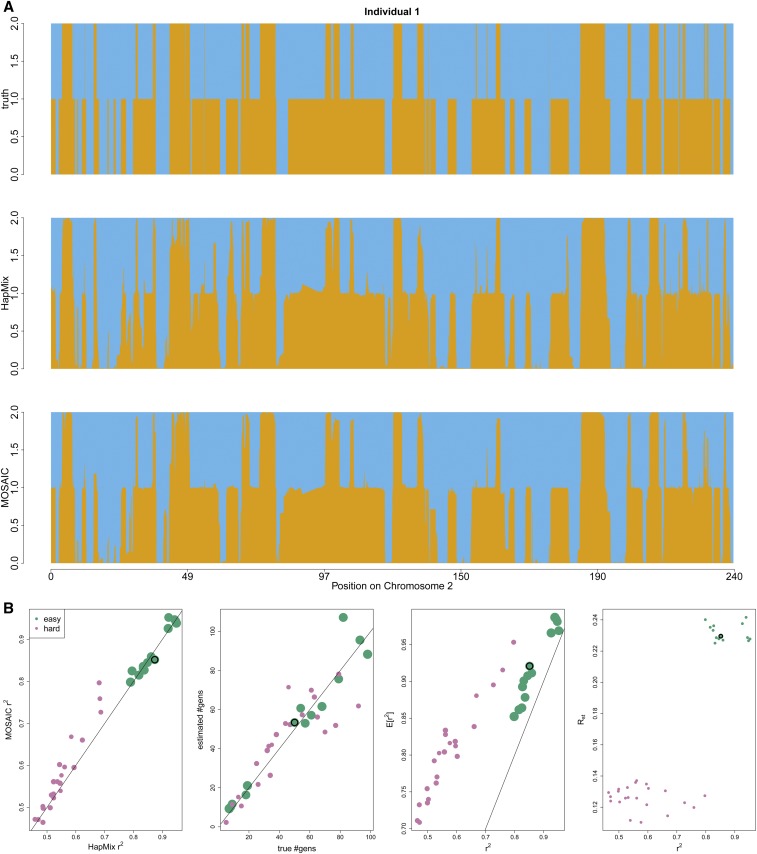
Comparison between MOSAIC and HapMix in two-way admixture simulations, as described in *Simulation Studies*. (a) Example diploid local ancestry (orange or blue) in a simulated dataset. Top is truth, middle HapMix, bottom MOSAIC where rephasing is as per *Rephasing*. Both methods infer similarly accurate local ancestry; however, MOSAIC is more confident, extends to more than two-way events, and does not depend on prior knowledge of mixing groups. (b) Easy (Yoruba and French) and harder (Pathan and Mongola) admixture simulations. Left: $r^{2}$ for HapMix against MOSAIC, showing superior local ancestry estimation against the current state-of-the-art in two-way admixture, even though HapMix is provided with known reference panels and MOSAIC is not. The plotting character is sized proportional to ${\text{R}}_{\text{st}}$. Left-center: true *vs.* inferred dates. Right-center: estimated coefficient of determination of local ancestry $E\left[r^{2}\right]$ (Equation 2) *vs.* squared correlation between true and estimated local ancestry. Right: ${\text{R}}_{\text{st}}$ (Equation 4) against squared correlation between true and estimated local ancestry, showing that ${\text{R}}_{\text{st}}$ can be used to identify challenging cases. In all plots, the black circle highlights the simulation shown in (a).

Examination of the ${\text{R}}_{\text{st}}$ value sheds light on why $E\left[r^{2}\right]$ is low; uncertain local ancestry estimates evidenced by a low $E\left[r^{2}\right]$ could be due to one or more of the following reasons: low divergence between mixing groups, inadequate panels, a long time since admixture, a very minor contribution of one of the mixing groups; a value of ${\text{R}}_{\text{st}}$ that is low points toward the utility of the panels. For this reason, we recommend examination of ${\hat{F}}_{\text{st}},{\text{R}}_{\text{st}}$, and $E\left[r^{2}\right]$. For example, Figure 2b demonstrates that ${\text{R}}_{\text{st}}$ identifies cases where the panels do not contain good surrogates for the mixing groups (green points) rather than the admixture event being too old to clearly quantify.

Finally, it should be noted that, when there is no clear admixture signal, MOSAIC sometimes returns very small estimated minor ancestry proportions, and, in this case, the estimated ${\text{F}}_{\text{st}}$ between the latent ancestries is meaningless. All segments are assigned to one ancestry, and the Weir and Cockerham estimator breaks down. This occurred for four model runs of the extended 95 population Human Genome Diversity Project (HGDP) dataset (see *Application to human genome diversity project data*), all for two-way admixture models; Germany-Austria (GerAus), Karitiana, Lahu, and Welsh. It is noteworthy that GLOBETROTTER also found no strong evidence of admixture for these populations.

### Dating admixture events using coancestry curves

We wish to infer the times at which each pair of ancestral groups admixed, based on our model fit. The transition rates matrix Π does not provide a direct estimate of these times (in number of generations), and so we rely on construction of coancestry curves as per Hellenthal *et al.* (2014) with best-fit exponential decay curves to estimate dates. In order to create the coancestry curves (in black lines in Figure 1c) and to find the best fit exponential curve (green lines), we first estimate the probability of being in ancestry *a* at one position and ancestry *b* at a position *d* away, *relative to genome-wide average* probability. This naturally depends on how many generations have occurred since the admixture event. As shown in the Supplement to Hellenthal *et al.* (2014) Equation S11, for two positions $x_{1}$ and $x_{2}$ that are *d* apart, this relative probability P(a,b,d) is equal to $\delta_{ab}e^{-d\lambda_{ab}}+\tau_{ab}$, assuming a single admixture event. Here $\tau_{ab}$ is the asymptotic value and thus is known to be ∼1 as it is the ratio of the probability of being ancestry *a* at any locus and ancestry *b* at an unlinked locus to the genome-wide average. $\lambda_{ab}$ is the generations since the admixture, and we scale *d* by grid width so that $\lambda_{ab}$ is the number of generations.

We therefore find constants $\delta_{ab},\tau_{ab},\lambda_{ab}$ that minimize the squared difference between P(a,b,d) and this exponential curve, averaging over all pairs of points these distances *d* apart. We find that the numerical optimization is relatively unstable, so we need to initialize with sensible $\delta_{ab},\tau_{ab},\lambda_{ab}$ values. We note that $\tau_{ab}≃1$, at d=0 we have $\delta_{ab}=P\left(a,b,0\right)-\tau_{ab}$ and at any nonzero distance *d* (*e.g.*, where the height of the curve is halfway between the height at 0 and its asymptote)

$$\lambda_{ab}=\frac{-\text{log}\left(\frac{P\left(a,b,d\right)-\tau_{ab}}{\delta_{ab}}\right)}{d}.$$

Thus, we have crude estimates of $\delta_{ab},\tau_{ab}$, and $\lambda_{ab}$ with which to start a numerical optimization routine, which derives the best fit (green lines). Although Figure 1c demonstrates curve fitting by imposing the assumption that $\lambda_{ab}$ is the same for all values of *a* and *b*, we proceed all subsequent analysis with this assumption relaxed and dates for events are the average across a,b.

An assumption in MOSAIC is that of a single admixture event for each pair of ancestries in each individual. Where this assumption is not met, the coancestry curves will not be well approximated with a single exponential decay as above. The alternative is that of either multiple waves of admixture or continuous gene flow between diverged populations. These latter two are difficult to distinguish using these coancestry curves [see S2.4 of the Supplement to Hellenthal *et al.* (2014)]. The single-exponential curve fit can, in principle, be used to determine whether the assumption of a single event should be rejected, but, although visual inspection may suggest evidence of multiple admixture times, MOSAIC does not provide a formal test for this. Figure S1 of the Supplement demonstrates highly accurate inference of admixture dates based on simulations for both single-date and flexible-date versions of this approach.

### Interpretation of pairwise decay parameters for multiway admixture

Examination of the pairwise coancestry plots does not always yield a unique inference of the demographic history of the admixed population. For an A-way admixture model there are A−1 events if we assume each population mixes with the already admixed population that includes all previously separate populations; however, each event does not necessarily involve mixture between the inferred ancestral groups. For example, in a three-way admixture between groups a,b, and c (without loss of generality) the history of the admixed abc samples could have arisen via several possible admixture sequences:

1. a+b+c (single event).
2. a+b then ab+c (two events).
3. a+b then b+c then ab+bc.

Other more complex histories are also possible involving combinations of continuous and event based admixture. We will restrict our focus to sequence types 1 and 2 above, that is we will only consider cases wherein each ancestral population mixes just once with the admixed group. We will also restrict focus to admixed populations in which the individuals all share an approximately common history. This precludes for example a+b then b+c then a+c (three events and no abc admixed individuals).

If we *knew* pairwise dates to be $\lambda_{ab}=\lambda_{ac}=\lambda_{bc},$ we might infer that the history is of sequence 1 above (it could also be that there were simultaneous but separate events occurring). If we infer pairwise dates to be $\lambda_{ab}>\lambda_{ac}$ and $\lambda_{ac}=\lambda_{bc},$ the interpretation would be that a and b mixed followed by ab mixing with population c (as per sequence 2 above). However, where $\lambda_{ab}>\lambda_{bc}>\lambda_{ac},$ we would infer that the events were sequential and nonoverlapping. In such cases the inference is that a and b mixed followed by a mixture involving unadmixed (with respect to a) individuals of ancestries b and c, followed by a third event involving admixture between a and c only (without b). This is of course possible if we do not observe individuals inferred to have all three ancestries occurring on their genome. Sequence 3 is less straightforward to infer; $\lambda_{ab}$ will depend on tracts with a length distribution based on a mixture due to a and b admixing twice (with and without the presence of c).

We do not know the pairwise *λ* values, but estimate them using the coancestry curves; uncertainty in which history we should infer is confounded with the presence of multiple waves of admixture or continuous gene flow and manifests as bootstrapped *λ* values that are highly variable. For example, sequence 3 will give rise to $\lambda_{ab}$ values indistinguishable from those induced by two pulses of admixture between a and b. For these reasons, we do not claim to have made a definitive contribution to the reconstruction of admixture histories based on local ancestry estimation but simply interpret our multiway admixture results in this context.

### Data availability

An open source R package is available for download at https://maths.ucd.ie/∼mst/MOSAIC/. A browser for all results on the extended HGDP dataset (from Hellenthal *et al.* 2014; see *Application to human genome diversity project data*) is also provided. The data are publicly available at https://www.ncbi.nlm.nih.gov/geo/query/acc.cgi?acc=GSE53626 as per Hellenthal *et al.* (2014). Supplemental material available at FigShare: https://doi.org/10.25386/genetics.8144159.

## Results

### Simulation studies

Figure 1, b and c depicts a simple simulation study. We simulated admixture 50 generations ago using real haplotypes from French and Yoruban chromosomes in a 50-50 split; thus the ground truth local ancestry is known. Panels are then formed from Norwegian, English, Ireland, Moroccan, Tunisian, Hadza, Bantu-Kenya (BantuK), Bantu-South-Africa (BantuSA), and Ethiopian individuals. MOSAIC inferred the stochastic relationships between these groups and the underlying mixing groups, along with all other parameters using the algorithm detailed in *Algorithm*. As can be seen from Figure 1c, MOSAIC is able to accurately infer the correct simulated admixture date. Under this simple simulation, the algorithm was also able to infer that one ancestry heavily copies from the West European populations and the other from African populations.

The current state-of-the-art in local ancestry estimation in the context of a two-way admixture is provided by HapMix (Price *et al.* 2009). We therefore also ran HapMix in haploid mode with EM to learn its model parameters, and then performed a diploid (integration over all possible phasings) run to estimate diploid local ancestry. Reference panels were necessarily provided to HapMix in two sets of proxy haplotypes for the two mixing groups (European and African). This took 50 min 5 sec, in comparison with the total MOSAIC run time of 29 min 8 sec (on a standard laptop, which included the time taken to sample the simulated data, fit coancestry curves, etc.) for full genomes. Figure 2a shows the true and estimated local ancestries under both models along chromosome 2 for a single diploid individual.

We next report performance of MOSAIC on scenarios with various levels of difficulty, and again compare with HapMix. We fit both models to simulations similar to the above, but for varying admixture dates and for a second mixture; the latter is a mixture of Pathan and Mongolian genomes, and the reference panels used are Iranian, Lezgin, Armenian, Sindhi, Brahui, Georgian, Hezhen, HanNchina, Han, Tu, Oroqen, Daur, Xibo, Tujia, and Yakut. Again, these must be provided to HapMix as two sets of known surrogates, whereas MOSAIC infers the relationships to the admixed genomes. See Figure 2b for a summary of MOSAIC’s and HapMix’s performances across these scenarios. MOSAIC typically outperforms HapMix in terms of $r^{2}$ with the true local ancestry, especially for the more difficult scenarios.

Figure 2b (right-center) demonstrates that $E\left[r^{2}\right]$ provides a useful predictor of local ancestry accuracy based upon the MOSAIC output; however, it overestimates $r^{2}$ with the level of bias depending on the genetic divergence between the mixing groups and the reference panels (as measured by ${\text{R}}_{\text{st}}$). This bias is expected, as $E\left[r^{2}\right]$ estimates squared correlation between the inferred local ancestry (which is continuous) and the true local (which is 0,1, or 2 for diploid ancestry), assuming that true ancestries are sampled from the inferred ancestry, *i.e.*, that the model fit, and, hence, the conditional ancestry probabilities, are accurate overall. Hence, modeling departures or model misidentification are expected to result in overestimation of confidence.

It must be stressed here that this is a setting that is ideal for HapMix (two-way admixture with known, highly appropriate reference panels), whereas MOSAIC generalizes to multi-way admixture, and can return useful results when the reference panels are not good surrogates for the mixing ancestral groups. Furthermore, MOSAIC infers the stochastic relationships between panels and ancestries. Further demonstration of the robustness of MOSAIC to imperfect reference panels is shown in Section S4 for two-way admixture; Figure S4 demonstrates inference of admixture within reference panels in terms of copying matrix values μ, along with accurate date of admixture estimates for a simulation involving admixture between Spanish and Yoruban genomes, but with reference panels from only continental Africa. Table S3 shows that ${\text{F}}_{\text{st}}$ between donor panels and the panels used to simulate admixed genomes is also accurately estimated from the partially reconstructed ancestral genomes, leading to the conclusion that MOSAIC can accurately estimate ${\text{F}}_{\text{st}}$ between modern populations and ancestral groups for real events.

We also verified the excellent performance of MOSAIC by performing three-way simulations of admixed individuals with equal ancestry from French, Mandenkan, and Han Chinese populations, which were then hidden as donors. For full details, see Section S5 of the Supplemental Material. Across admixture times from 5 to 100 generations ago, we evaluated the performance of MOSAIC and the methods ELAI, LAMP-LD, and RFMix, which can handle multi-way admixture. We varied the donor groups available to detect ancestry segments, including simulations wherein one of the reference panels is created with admixture similar to the target genomes (Figure S6 and Section S5.1). MOSAIC performed extremely well for cases with recent admixture (five generations), particularly when the donor groups were similar to the true admixing groups, but still captured >75% of the information regarding ancestry segments, even for events 50 generations ago (Figure S5 and Tables S4 and S5). Moreover, for every simulation scenario, MOSAIC uniformly outperformed all of the alternative approaches, even though these alternative approaches were provided with parameter values (as necessary) chosen to match those of the simulations, and were also provided with panels most closely representing each respective ancestry, among those available. In contrast, MOSAIC inferred all properties of the underlying latent ancestries and other model parameters using the data.

### Application to human genome diversity project data

We reanalyze the same 95 population dataset in Hellenthal *et al.* (2014), which is an extended version of the HGDP. For details on these populations see Table S6.1 of the Supplement to Hellenthal *et al.* (2014).

### Simple two-way admixture analysis

**Figure 3:**
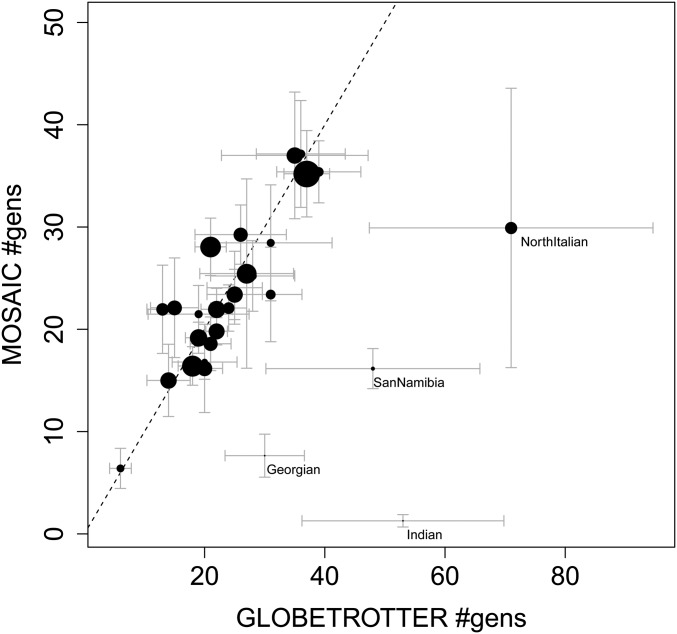
Inferred dates from MOSAIC are plotted against inferred dates from GLOBETROTTER, including bootstrapped ±2 SE bars for real data two-way admixture events (see note in *Simple two-way admixture analysis*). The GLOBETROTTER dates are from table S14 of Hellenthal *et al.* (2014). The size of the each disc of each event is proportional to ${\text{R}}_{\text{st}}$ (see *F_st_ summaries*). For a table of estimated and bootstrapped intervals see Table S1.

MOSAIC handles multiway admixture and provides accurate local ancestry inference; however, we first restrict to the case of two-way admixture events in order to compare results with the current state-of-the-art method GLOBETROTTER (Hellenthal *et al.* 2014). This method infers admixture proportions and timings without specification of surrogates for the mixing groups; however, it does not estimate local ancestry. Figure 3 shows the inferred dates for the target populations that were found to have experienced a single two-way admixture event according to Hellenthal *et al.* (2014). The *x*-axis shows the inferred dates ± 2 SE (in generations since admixture) and the *y*-axis shows the MOSAIC inferred dates.

We do not propose an explicit model comparison method for selecting the number of ancestries but advise careful comparison of results for various models, as any fit will depend upon which panels were available. Where MOSAIC returns low $E\left[r^{2}\right]$ and/or low ${\text{R}}_{\text{st}}$, or if other parameter estimates are not compatible with an interpretable model fit (such as inferred generations since admixture being <1), we advise fixing on a lower number of ancestries. Section S1 provides additional details on these two-way results. Although the results in this section are presented only for two-way admixture, we include three- and sometimes four-way results for all 95 populations at http://maths.ucd.ie/∼mst/MOSAIC/HGDP_browser/.

The GLOBETROTTER paper creates bootstrapped chromosomes, and finds the sample distribution of dates based on these—a protocol we follow here. Although MOSAIC models a single admixture event that may be experienced at different times by different admixed individuals, here, we fit a set of coancestry curves with a common rate parameter for consistency with Hellenthal *et al.* (2014) and to provide a direct comparison. In Figure 3, most date estimates have ∼95% confidence intervals intersecting the line y=x, so are consistent between the two approaches. MOSAIC provides tighter bootstrapped confidence intervals, on average, but we observe that there are three cases for which MOSAIC infers a far more recent admixture with very narrow confidence intervals (bottom right of Figure 3), and several warning flags are raised when we analyze the output from MOSAIC for these three populations for a two-way admixture event.

The ${\text{R}}_{\text{st}}$ statistic (which is based only on the MOSAIC results) is smallest for the target populations that have the strongest disagreement with GLOBETROTTER (Georgia, San-Namibia, and India; labels appear below and to the right of plotting points in Figure 3 for these populations). For India, Sindhi is the closest (smallest ${\hat{F}}_{\text{st}}$) to both admixing groups. Similarly, for San-Namibia, the San-Khomani are the best match to both ancestries. In the Georgian case, the Armenians and Russians are extremely close in ${\hat{F}}_{\text{st}}$ to both ancestries. GLOBETROTTER is more suited to very old admixture events, and does not require the ability to infer admixture breakpoints along the genome. In the GLOBETROTTER analysis of India, the major ancestry is represented by South Central Asian populations (Sindhi, Pathan, Indian Jew); however, the minor ancestry (14%) is highly diverse, consisting of Asian populations (Cambodian, Mongola, Han) as well as Ethiopian and Papuan. We find a similar $E\left[r^{2}\right]$ for two- and three-way admixture (0.604 and 0.601, respectively), although neither exhibit a clear admixture signal as measured by the ${\text{R}}_{\text{st}}$ statistic (0.0045 and 0.016, respectively).

Note that the bootstrapped SE for the dates in Figure 3 are created using a single rate parameter estimate, whereas the plots shown here estimate one such parameter for each unique unordered pair of ancestries (*i.e.*, 1−1,1−2,2−2 for two-way admixture). For a three-way (or higher) model, a single date may be invalid, and we therefore provide one such coancestry plot for each pair of ancestries in Figure 5 (see *Interpretation of pairwise decay parameters for multiway admixture*).

**Figure 4:**
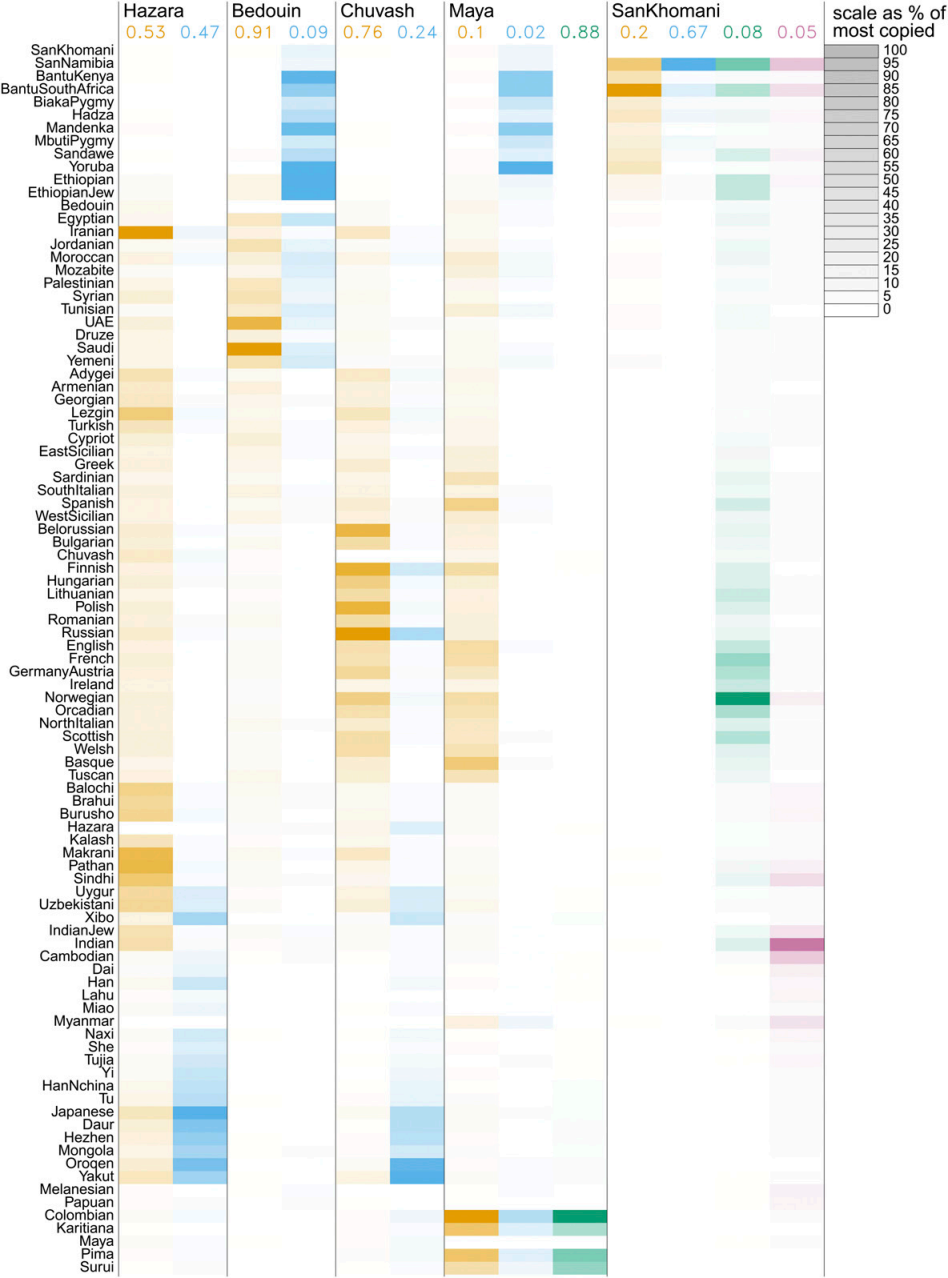
Inferred copying matrices for case studies of human admixture based on the HGDP dataset. The copying proportions $\mu_{pa}$ are scaled within columns to % of the most copied donor population so that each cell shading is equal to $100.\mu_{pa}/\text{arg}{\text{max}}_{p}\mu_{pa}$. Along the top are the estimated genome-wide ancestry proportions averaged across all admixed target individuals, *e.g.*, Hazara are 53% “orange” ancestry.

**Figure 5:**
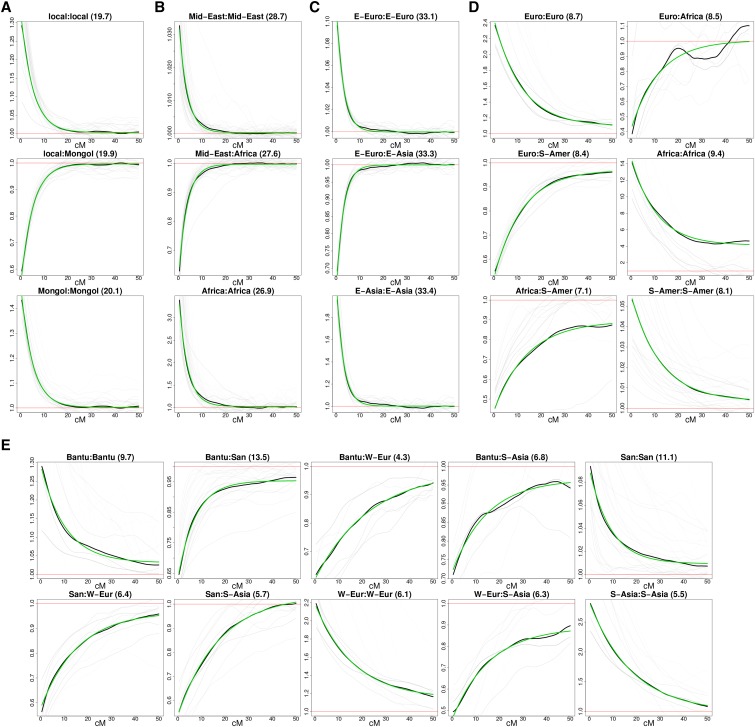
(a) Hazara two-way. (b) Bedouin two-way. (c) Chuvash two-way. (d) Maya three-way. (e) SanKhomani four-way. Coancestry curves for case studies of admixture within the HGDP dataset. On the top of each subplot, the ancestry sides are labeled according to the closest donor panel as measured by ${\hat{F}}_{\text{st}}$ (see Table 1, Table 2, Table 3, Table 4, and Table 5) and the estimated number of generations since admixture between each pair of ancestries is given in brackets. The relative probability of pairs of local ancestries (*y*-axis) as a function of genetic distance (in centi-Morgans, *x*-axis) is shown in black (across all individuals), gray (one per individual), and fitted exponential decay curve (green). The departure from the exponential decay seen in the top right panel is due to the low African (2%) and European (10%) ancestry dosages coupled with the recent admixture date, meaning that local ancestry switches between these two are rare. This low number of switches results in a noisy decay curve for each individual (gray lines).

We now turn our attention to a number of case-studies, specifically Hazara, Bedouin, and Chuvash two-way, Maya three-way, and San-Khomani four-way admixture models, to understand how MOSAIC performs relative to previous analyses of these groups. Figure 4 depicts the inferred copying matrix μ for each of these case-studies, and Figure 5 shows the coancestry curves used to date the events.

### Hazara two-way admixture

**Figure 6:**
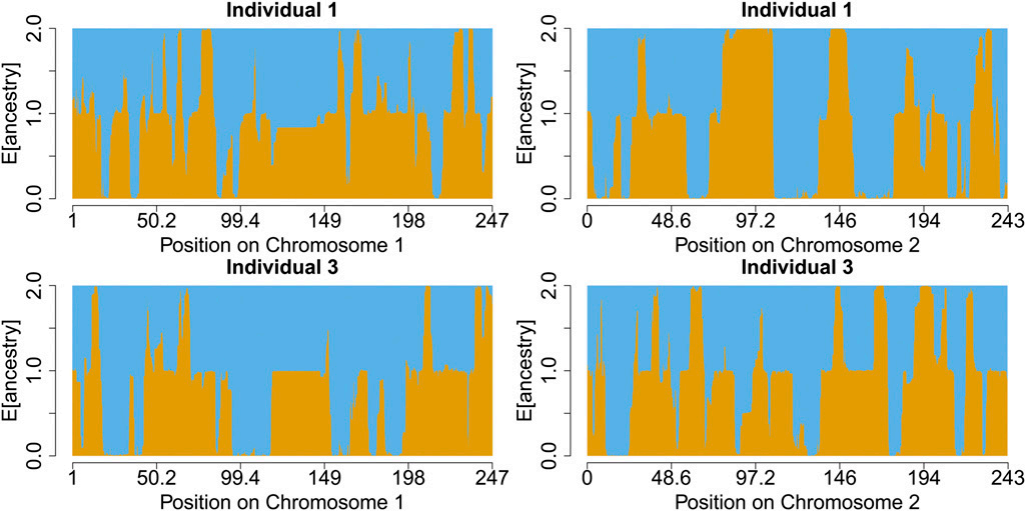
Hazara estimated local ancestry on chromosomes 1 and 2 across two individuals. There is a roughly 50–50 ancestry contribution from two sources ∼20 generations ago. The orange source is Pathan like and the blue is Mongolian like (see Table 1 for details). The colors are consistent with Figure 4, which shows scaled copying proportions for each donor panel.

Hellenthal *et al.* (2014) find that the Hazara—an ethnic group mainly living in Afghanistan—“show the clearest signal of admixture in the entire dataset”; this is reflected by MOSAIC inferring tightly coupled coancestry curves (Figure 5a), with highly confident local ancestry estimates (Figure 6). MOSAIC identifies the two admixing groups as close matches to the modern-day Pathan and Mongola (47%) populations (Table 1), confirming the likely Mongol origin admixture with local Iranian-like populations in this group (*e.g.*, Hellenthal *et al.* 2014). We have chosen to include local ancestry estimates for two individuals along two chromosomes, but these are representative of the entire population of 22 individuals.

**Table 1:** ${\text{F}}_{\text{st}}$ estimates between local ancestries and the closest five panels in Hazara two-way

Pathan	0.0066	Mongola	0.0075
Iranian	0.007	Xibo	0.0084
Turkish	0.0078	Daur	0.0089
Balochi	0.0089	Oroqen	0.01
Sindhi	0.0089	Hezhen	0.011

${\hat{F}}_{\text{st}}$ between the inferred local ancestries is 0.087.

### Bedouin and Chuvash two-way admixture

The Bedouin (a nomadic Arab group in North-East Africa/Arabian peninsula) are fit by MOSAIC as a two-way admixture between groups most closely related to groups from the Middle-East (91%, see Figure 4) and present-day sub-Saharan African groups (Table 2), ∼28 generations ago (Figure 5b). In *Possible selection signal at the HLA in North Africa*, we further discuss results of applying MOSAIC to these 45 Bedouin individuals along with other groups from North Africa that show similar admixture structure.

**Table 2:** ${\text{F}}_{\text{st}}$ estimates between local ancestries and the closest five panels in Bedouin two-way

Saudi	0.007	BantuK	0.025
Jordanian	0.0076	Yoruba	0.031
Syrian	0.0082	BantuSA	0.033
Palestinian	0.0093	Mandenka	0.034
Cypriot	0.0095	Sandawe	0.034

${\hat{F}}_{\text{st}}$ between the inferred local ancestries is 0.13.

The Chuvash population were previously analyzed after removing several distinct Eastern European donor populations Hellenthal *et al.* (2014), due to somewhat similar admixture events in the removed groups. The MOSAIC analysis of 16 Chuvash individuals (${\text{F}}_{\text{st}}$ Figure 5c and Table 3) identified a signal of admixture between European and East Asian (24%) populations at an estimated admixture date of ∼33 generations ago, broadly corroborating previous results Hellenthal *et al.* (2014), without requiring the removal of such samples, supporting the robustness of MOSAIC to admixed panels. We also fitted a three-way admixture model (Section S2.2 of Supplement), which again showed 900-year-old admixture from East Asian groups, but also suggested additional complexity involving ancestry from Caucasus-like (15% of the global Chuvash ancestry), East-Asian like (24%) and East-European populations, at different times, including more recently.

**Table 3:** ${\text{F}}_{\text{st}}$ estimates between local ancestries and the closest five panels in Chuvash two-way

Russian	0.0046	Oroqen	0.032
Polish	0.0048	Yakut	0.033
Belorussian	0.0051	Mongola	0.036
Hungarian	0.0055	Xibo	0.037
GerAus	0.0057	Daur	0.038

The ${\text{F}}_{\text{st}}$ estimate between the inferred local ancestries is 0.11.

### Maya three-way admixture

The Maya are a Central American population that exhibit recent (17th century) three-way admixture between European (10%), African (2%), and Native American (88%) groups in the 21 individuals analyzed here, according to our analysis. The $E\left[r^{2}\right]$ values for two-way (0.96) and three-way (0.957) MOSAIC runs are close, suggesting support for the three-way model. See Table 4 for modern populations that are closest in terms of ${\text{F}}_{\text{st}}$ to the mixing groups. As per Hellenthal *et al.* (2014), these results corroborate known colonial era migration from Spain and West Africa from the 15th and 16th centuries, and a single admixture event involving all three ancestries is compatible with the pairwise coancestry curve estimates in Figure 5d as they all heavily overlap for each pair of ancestries with uncertainty obtained via a bootstrap routine.

**Table 4:** ${\text{F}}_{\text{st}}$ estimates between local ancestries and the closest five panels in Maya three-way

Spanish	0.014	BantuK	0.029	Colombian	0.035
Romanian	0.015	Yoruba	0.032	Pima	0.057
French	0.016	Mandenka	0.035	Karitiana	0.077
Tuscan	0.016	Sandawe	0.036	Hazara	0.099
Bulgarian	0.016	BantuSA	0.036	Uygur	0.1

The ${\text{F}}_{\text{st}}$ estimate between the inferred local ancestries is 1 × 2 = 0.17 1 × 3 = 0.18 2 × 3 = 0.29.

### San-Khomani four-way admixture

As per Choudhury *et al.* (2017), we find evidence of four-way admixture between Bantu-speakers, Khoesan, Europeans, and populations from southern Asia in San-Khomani individuals from Southern Africa. Analyzing 30 individuals, we obtain $E\left[r^{2}\right]=0.896$ for a four-way analysis with the minor ancestry contributing 5%. Choudhury *et al.* (2017) explained that the lack of availability of whole genome sequencing data for (unadmixed) Khoesan source populations restricted their ability to perform analyses such as admixture mapping and local ancestry inference. A strength of MOSAIC is that it can leverage haplotypes from particular ancestry sources embedded within admixed donor genomes (for example Khoesan ancestry haplotypes within admixed San-Namibia population genomes), to overcome this challenge. Further, the ${\text{F}}_{\text{st}}$-based analysis (see Table 5) is able to separate the actual haplotypes involved so that a clear disambiguation between all four ancestry components is achieved.

**Table 5:** ${\text{F}}_{\text{st}}$ estimates between local ancestries and the closest five panels in SanKhomani four-way

BantuSA	0.0066	SanNamibia	0.02	French	0.011	Uygur	0.024
BantuK	0.01	BiakaPygmy	0.09	Welsh	0.011	Uzbekistani	0.025
Yoruba	0.012	BantuSA	0.09	Bulgarian	0.012	Sindhi	0.025
Mandenka	0.018	Sandawe	0.099	Romanian	0.012	Hazara	0.026
Sandawe	0.022	MbutiPygmy	0.11	Spanish	0.012	Indian	0.027

The ${\text{F}}_{\text{st}}$ estimate between the inferred local ancestries is 1 × 2 = 0.1 1 × 3 = 0.14 1 × 4 = 0.14 2 × 3 = 0.27 2 × 4 = 0.27 3 × 4 = 0.066.

**Figure 7:**
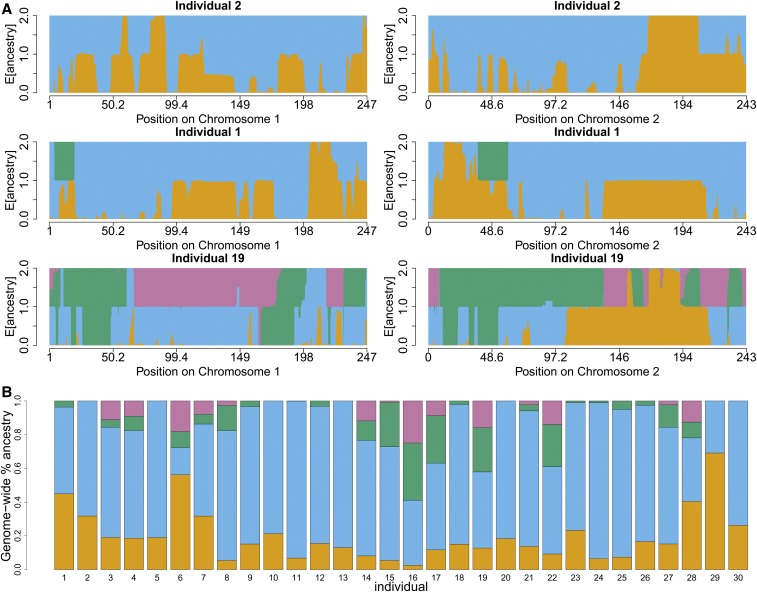
Details of San-Khomani four-way admixture model fit. Each color represents one of four latent ancestries, which in this case correspond to four different ethnicities. The orange source is Bantu-like, blue is San, green is European, and purple is Asian (see Table 5 for details). The colors in these plots are consistent with Figure 4, which shows scaled copying proportions for each donor panel. On Figure 4, the San-Khomani column gives the genome-wide proportion (across all 30 analyzed individuals) of these four ancestries, and this figure shows ancestry proportions per individual as stacked bar plots. (a) San-Khomani estimated local diploid ancestry dosage (four colors) on chromosomes 1 and 2 across two individuals. The first individual has no inferred “Asian” like ancestry (see Table 5), but the other two do. (b) Diagram showing the inferred proportions of the three ancestries in the San-Khomani.

Figure 7a illustrates the local ancestry estimation for three individuals chosen to have markedly different ancestry components:

2 two-way admixture between Bantu 32% and Khoesan 68%.
1 three-way admixed Bantu 45%, Khoesan 51%, and European 4%.
19 four-way admixed Bantu 13%, Khoesan 45%, European 26%, and Asian 16%.

This illustrates the flexibility of the method, as it successfully infers the variable mixing proportions across individuals in a single model fit.

Figure S2b of the Supplement shows the sample density over 500 bootstrap samples of the estimated dates. From this, we infer that, for the San-Khomani, the estimated chronological order of pairwise events are (Bantu + San), (San + West-Europe), (San + South-Asia), (West-Europe + South-Asia), (Bantu + South-Asia), and (Bantu + West-Europe). In this case, there is heavy overlap between all but the oldest event although the high degree of uncertainty surrounding (Bantu + South-Asia) means that this event does overlap it. The simplest explanation for this is that Bantu and San like ancestors mixed first 13.5 generations ago, followed by admixture with all other groups from ∼10 generations ago onwards.

### Online results browser

Details for results of applying MOSAIC analysis to 95 global populations, including those shown above, are available at http://maths.ucd.ie/∼mst/MOSAIC/HGDP_browser/. Highlights include the presence of a small but detectable signal of 1–3% Central Asian ancestry in Eastern European populations. *e.g.*, in Belorussian (2%), Hungarian (2%), Bulgarian (3%), Romanian (3%), and Polish (1%) in a two-way admixture with North-West European groups the major ancestral component. Admixture appears to have occurred between ∼24 and 35 generations ago, with the central Asian groups most similar to modern day Uzbek, Uygur, and Hazara groups. The Bulgarian and Romanian results show support for three-way admixture as they have similar (or higher) $E\left[r^{2}\right]$ values for two-way and three-way models. In these cases, the results suggest Southern European (54.5%), North and West European (43%), and Central Asian (2.5%) ancestries. Section S6 provides results on three additional case studies, namely Moroccan two-way, Chuvash three-way, and North African two-way. A number of populations cause MOSAIC to issue a warning that there may be no admixture signal detected (for the analyzed samples using the available panels). For example, Ireland and Scotland results in less than one generation since admixture, with no detectable difference between mixing groups (${\hat{F}}_{\text{st}}$ and ${\text{R}}_{\text{st}}$ both zero). Germany-Austria and Welsh results in all individuals having estimated minor ancestry proportions of zero.

### Possible selection signal at the human leukocyte antigen region in North Africa

**Figure 8:**
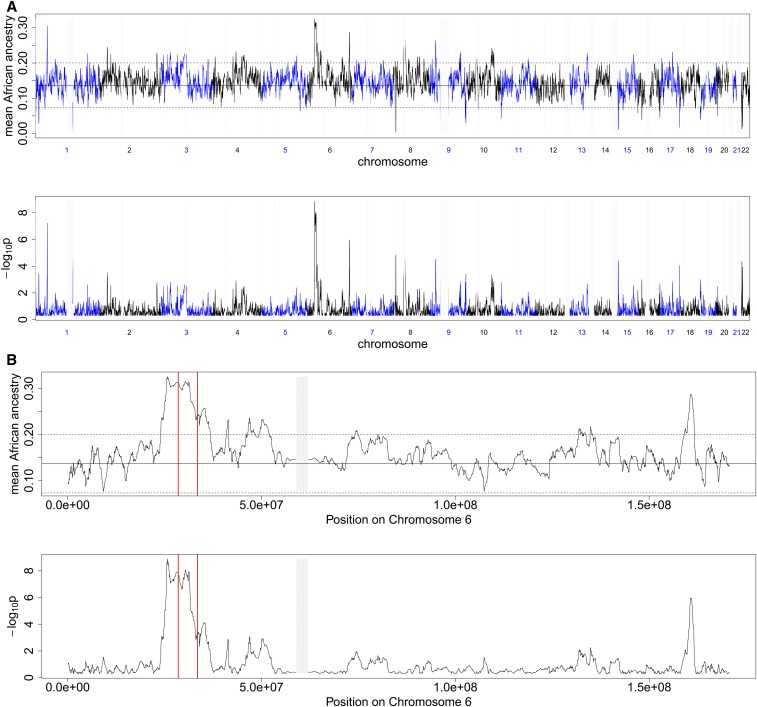
Mean African Ancestry and -log_10_p of mean ancestry across all 220 individuals in North Africa plotted against: (a) genome position (b) Chromosome 6 position. There is a high and wide spike at the HLA (marked by two vertical red lines) on Chromosome 6 at the HLA. Note that we have blocked out (in light gray) all 1 Mb regions with <10 markers; this includes centromeres with low recombination rates and few SNPs.

If particular ancestral backgrounds are associated with adaptively beneficial alleles, then, following admixture, we expect the average population proportions of such ancestries to rise nearby, producing peaks in average ancestry (see Figure S12 and Section S8 of the Supplement for simulations of such a scenario). To examine this in practice, we explored a region of North Africa and the Middle East, collectively possessing a sample size of 220 individuals with a proportion of sub-Saharan ancestry (see Section S6.3 of Supplement for additional details), derived from admixture events which we date to ∼31 generations ago, sufficient for such selection to plausibly occur. Identifying ancestry segments by including European-like and sub-Saharan African groups in a MOSAIC analysis, we observe a genome-wide significant peak of African-like ancestry around the human leukocyte antigen (HLA), the strongest such signal in the genome (Figure 8). Other possible selection signals are observed (*e.g.*, on chromosome 1) but these correspond to narrow spikes relative to the inferred admixture date, and, so, rather than postadmixture selection, are more likely to reflect more ancient events. The HLA is a gene complex that includes many proteins responsible for regulation of the immune system, and are, therefore, a region upon which natural selection is believed to act; indeed HLA loci are identified to be among the fastest evolving in the human genome (de Bakker *et al.* 2006). Altered ancestry proportions in the HLA have been inferred previously in Mexican individuals (Zhou *et al.* 2016), and the HLA shows an apparent excess of identical-by-descent (IBD) sharing (Botigué *et al.* 2013), but whether such patterns truly reflect selection remains controversial (Price *et al.* 2008) due to complex LD patterns in the HLA, for example, due to ancient balancing selection (de Bakker *et al.* 2006). Additionally, unusual linkage structure and increased variation at the HLA has been linked to balancing selection (de Bakker *et al.* 2006). To test whether LD patterns within the HLA itself might explain the observed patterns, we excluded all SNPs within this region and reapplied MOSAIC (see Figure S9 and Section S7.1), which still showed a clear peak, implying a broad-scale signal, consistent with postadmixture selection toward African ancestry.

We further tested robustness of the signal by expanding our reference panel to include all groups not directly considered for admixture here. Strikingly, the peak at the HLA was eliminated (Figure S10 and Section S7.2), due to a number of haplotypes of previously uncertain but African-like ancestry having similar copies in southern European groups. This sharing of haplotypes between North Africa and southern European countries (which also have low levels of African ancestry) implies that, if the peak in inferred ancestry truly reflects selection for African ancestry in the HLA, this selection extends across the Mediterranean. In any case, it implies the extreme, rapid spread of similar haplotypes across a wide geographic region, at the HLA.

This increased selection of HLA types from the minor (African) ancestry could be explained either as an example of positive selection due to African HLAs being more effective in (for example) dealing with African continent pathogens, or an example of balancing selection to advantageous immune response diversity (Bronson *et al.* 2013). This second hypothesis is possible, because the Sub-Saharan ancestry has a larger effective population size and is the minor contributor to the admixture event, both of which result in increased diversity with increased ancestry from this side. Further investigation of this event is, however, warranted; although MOSAIC is designed to be robust to the presence of admixture (with respect to the ancestral mixing groups) in the donor panels at a genome-wide level, if there is the same selection signal in the donors then the signal in the targets becomes masked. Finally, we simulated admixture 31 generations ago using the closest donor panels to the ancestral groups and the remaining panels were used in a MOSAIC fit. Were bias toward African copying at the HLA due to the linkage structure and increased variation, we would expect to see this manifest in plots of the average local ancestry; however, no such pattern was observed under repeated simulations (see Figure S13 and Section S8).

## Discussion

We have created freely available software that implements an efficient and highly accurate model for fitting multi-way admixture events. We call this method, and the associated software, MOSAIC. It can not only handle multi-way admixture events but, unlike all currently available methods, it infers the stochastic relationship between groups of potential donors to use in the Li and Stephens’ type HMM and the underlying ancestral groups. Thus, a close relationship between donor surrogate haplotype panels and ancestral groups is not required.

We have demonstrated that, even in the case of available and known direct surrogate donor panels for the mixing groups, we can more accurately estimate both local ancestry along the genome and the parameters governing the event (number of generations since admixture and proportion of mixing ancestries) than the current state-of-the-art approaches. We have presented results on selected case studies of admixture that include replication of known well characterized two-way admixture events as well as some novel multiway results. An online browser at http://maths.ucd.ie/∼mst/MOSAIC/HGDP_browser provides access to interactive viewing of a total of 95 global populations, each of which is analyzed using two-, three-, and (as required) four-way admixture models.

Finally, we have shown that MOSAIC can be used to detect and investigate postadmixture selection effects. Bias in across-individuals local ancestry toward the sub-Saharan like ancestry is found in North Africans at the HLA.

## Footnotes

1 The limit to how many sources we model is a computational one as we necessarily must sum over all possible ancestry pairs for each forward/backward pass of the algorithm at each gridpoint; thus the overhead scales as $O\left(A^{2}\right)$ where *A* is the number of ancestries we model.

2 We could sample ancestries along the genome, however using maximal ancestry assignment will perform a similar averaging along the genome faster.

3 We assign local ancestries when the probability is >0.8.

## Appendix: Computational Efficiencies

### Unique Haplotype Lists

In order to reduce the memory footprint of the model and algorithm, we store only the unique haplotypes at each gridpoint for the donors and target admixed genomes. These lists grow sublinearly with the number of samples and size of reference panels so that the method scales to huge datasets. We first read in the data SNP by SNP; each locus is assigned to the nearest gridpoint so that some gridpoints have several, some one, and some no observations. The emission probabilities simply require knowledge of the number of matches between any potential donor and each target and the number of observations of each at that gridpoint. This is stored efficiently in a table for fast lookup as required, *i.e.*, we require a table of all possible values of θX and (1−θ)(W−X), where *θ* is the estimated copying error rate, *X* is the number of mis-matches, and *W* is the number of observations. We enumerate all possible values of *X* and *W* once after compression and update the lookup table (which is modest in size, depending on the density of markers and number of individuals in the study) after each re-estimation of *θ* in our EM algorithm. A map from (donor, recipient, gridpoint) to this lookup table is also constructed exactly once to complete the compression to a grid.

### Thinning of Donors

The total number of hidden states in our two-layer HMM is A×N, where *A* is the number of admixing populations, and *N* is the total number of donor haplotypes. However, in practice, the forward-backward algorithm will place a non-negligible probability on only a small subset of the donors at any one position. We exploit this fact for computational efficiency as follows:

1. Fit the ancestry unaware single layer HMM using all potential donor haplotypes.
2. Use this to *rank* the donors *at each gridpoint* from highest to lowest forward-backward probability.
3. Record the vector of top $n_{g}$ such donors at each gridpoint for each haplotype such that over 99% of the probability is captured, up to a maximum of 100 donors.
4. Create the superset of the donors for both haplotypes of each individual at each gridpoint.
5. Use only these locally thinned set of donors at each gridpoint in the full two-layer ancestry aware HMM.

We use the top ranked donors for both haplotypes for each individual so that the thinning does not overly effect the phasing (see *Rephasing*), *i.e.*, both haplotypes can see and use the potential donors of the other haplotype belonging in each target individual. We find that using <100 of these local best donors gave results almost identical to allowing local copying from any of the full set of donors. For example, the median number of donors required to capture >99% of the copying probability in the ancestry unaware model was 66 for admixture simulated between French and Yoruban genomes, and 59 for admixture between French and English.

Thinning in this way means that the ancestry aware parts of the algorithm scale *independently* of the number of donors in the dataset. The thinning steps scale linearly with the number of donors and independently of the number of hidden ancestries, but these steps need only be performed several times in total (*Algorithm*). Once the HMM states have been so reduced, we need to track locally at each gridpoint which donors occur where in the thinned set, one gridpoint to the left (forward algorithm) and one gridpoint to the right (backward algorithm) as we need to calculate the probability of switches to the same haplotype. We therefore include two additional lists of donors at each gridpoint; the indices of the matching donors (should they appear in the top 100) to the left and to the right of each gridpoint.

### Computational Tricks for the HMM

In the forward (and backward) algorithms at each gridpoint, we need to compute the probability of switching from each hidden state into every other hidden state which is $O\left(A^{2}N^{2}\right)$. However, as per Fearnhead and Donnelly (2001) and Li and Stephens (2003), we do not need to examine all pairs of donor haplotypes. All switches are equally likely (given the latent ancestry switch status), except the nonswitch, where the same haplotype is copied for consecutive gridpoints. Thus, when examining the possible switches between gridpoint g−1 and *g*, we only need sum over all probabilities for copying any donor haplotype at g−1 and multiply our transition and emission probabilities by this with a small correction for self-switches.

### Initialization

To initialize our model, we first fit a single layer HMM that is ancestry unaware to the full set of donors genome-wide. This is similar to a gridded version of chromopainter (Lawson *et al.* 2012). We perform EM until convergence to estimate ρ,θ,M, where the latter is simply a vector of copying probabilities for each group.

We now wish to initialize values for the ancestry aware part of the model, *i.e.*, an M copying matrix with one column per hidden ancestry and the marginal probabilities of each ancestry *α*. We first impose windows of size 0.5 cM across the genome and calculate the expected number of switches into each donor group in each window. This window size is chosen so that there is typically one latent ancestry per window but multiple donor groups are copied from the number of generations undergoing recombination until we expect a single event in a window is 200. We then use EM to fit a mixture model where the number of mixtures is the number of hidden ancestries we wish to model. For haplotype *h* in window *w*, the expected number of switches ${\hat{S}}_{,w}^{\left(h\right)}$ into the panels is modelled as a mixture of Multinomials.
